# Immune checkpoint blockade and CAR T-cell therapy in T-cell/histiocyte-rich large B-cell lymphoma: Challenges and opportunities

**DOI:** 10.1016/j.heliyon.2024.e38023

**Published:** 2024-09-18

**Authors:** Taha Koray Sahin, Serkan Akin

**Affiliations:** Department of Medical Oncology, Hacettepe University Cancer Institute, Ankara, Turkey

**Keywords:** CAR T-cell, Immune checkpoint inhibitors, T-cell/histiocyte-rich large B-Cell lymphoma, Tumor microenvironment

## Abstract

T-cell/histiocyte-rich large B-cell lymphoma (THRLBCL) is a highly aggressive large B-cell lymphoma defined by a paucity of malignant B cells amidst a dense infiltrate of reactive T cells and histiocytes. The unique tumor microenvironment (TME) of THRLBCL, marked by extensive immune infiltration and high PD-L1 expression, poses significant challenges for immunotherapies. This review explores the therapeutic potential and resistance mechanisms of immune checkpoint inhibitors (ICIs) and chimeric antigen receptor (CAR) T-cell therapy in THRLBCL. While ICIs show promise due to the immune-suppressive nature of the TME, CAR T-cell therapy has demonstrated limited efficacy, often hindered by primary resistance and early relapse. Combining CAR T-cell therapy with ICIs and Bruton tyrosine kinase (BTK) inhibitors and developing novel CAR constructs targeting multiple antigens are potential strategies to enhance treatment outcomes. Further prospective studies are essential to corroborate these strategies and improve the prognosis for this challenging lymphoma subtype.

## Highlights

• Unique TME in THRLBCL challenges immunotherapy efficacy.
• CAR T-cell therapy shows limited efficacy in THRLBCL.
• Combining CAR T-cell therapy with ICIs may improve outcomes.
• Novel CAR constructs targeting multiple antigens offer promising strategies.

## Graphical abstract

**Figure:**
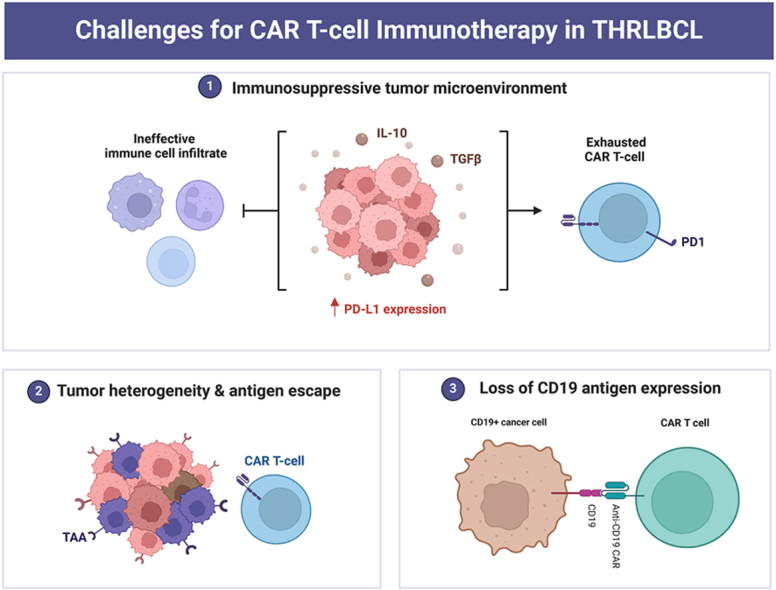

## 1 Introduction

T-cell/histiocyte-rich large B-cell lymphoma (THRLBCL) is a rare and highly aggressive subtype of LBCL, representing less than 5 % of cases [1]. Patients with THRLBCL tend to occur more frequently in younger male patients and advanced-stage disease compared to diffuse large B-cell lymphoma not otherwise specified (DLBCL) and is often associated with a grim prognosis [2]. Unlike the B-cell rich TME observed in DLBCL, THRLBCL exhibits a paradoxical composition characterized by a paucity of malignant B cells (<5 %) interspersed within an inflammatory background of reactive T lymphocytes and histiocytes, which present unique challenges in diagnosis and treatment [3]. Recent studies suggest that newly diagnosed THRLBCL patients may benefit from higher-intensity regimens, such as those based on R-CHOP-R-ICE, which have demonstrated improved survival and response rates [4]. However, while these higher-intensity regimens have improved clinical outcomes, they have not yet achieved the desired levels of efficacy. The atypical TME of THRLBCL has paved the way for studies exploring the potential of immunotherapies such as immune checkpoint inhibitors (ICIs) and chimeric antigen receptor (CAR) T-cell therapy in this lymphoma subtype. This review focuses on the current landscape of novel therapeutic strategies, the underlying resistance mechanisms, and paving the way for improved clinical outcomes in this challenging lymphoma subtype.

## 2 Overview of the tumor microenvironment and the potential of ICIs in THRLBCL

THRLBCL harbors a TME that differs from that observed in DLBCL. This unique niche is characterized by a complex interplay between immune cells, signaling pathways, and cytokines, fostering an immunosuppressive state that promotes tumor growth and progression [5]. A hallmark feature is the paucity of malignant B cells (<5 % of total cells) surrounded by a dense infiltration of reactive T cells and macrophages (histiocytes). This cellular composition significantly hinders the effectiveness of conventional chemotherapy in THRLBCL patients [6]. The gene coexpression network analysis has revealed that THRLBCL exhibits a pronounced interferon (IFN)-driven inflammatory pathway and PD-L1 signaling, which are crucial in modulating immune response within the TME [7].

A defining feature of the THRLBCL TME is the overexpression of the PD-L1/PD-1 immune checkpoint axis, representing one of the mechanisms well described in immune evasion and T-cell exhaustion [7,8]. The upregulation of programmed cell death protein-1 (PD-1) and PD-L1 genes, along with other immune checkpoint genes such as cytotoxic T-lymphocyte antigen 4 (CTLA-4) and lymphocyte activation gene-3 (LAG3), suggests a profoundly immune-suppressive microenvironment [7]. This is further corroborated by the presence of T cell–specific transcription factor 1 (TCF1^+^) progenitor exhausted T cells, a subset associated with better response to anti-PD-1 therapy, which are more abundant in THRLBCL than in DLBCL [9]. Panayi et al. further explored the complex immune checkpoint landscape of THRLBCL, revealing a significant expansion of PD-1 expressing CD8^+^ cytotoxic T lymphocytes and CD163+ macrophages, with a subset of the latter co-expressing PD-L1 [10]. This observation suggests the potential involvement of myeloid-derived suppressor cells (MDSCs) within THRLBCL TME, contributing to the overall immunosuppressive milieu. Taken together, these findings highlight that ICIs could be a promising treatment for THRLBCL.

Recently, Griffin et al. revealed that aggressive THRLBCL cells are characterized by a dense infiltration of PD-L1-expressing macrophages and T cells [8]. Following these findings, they evaluated the clinical efficacy of anti-PD-1 monotherapy in five THRLBCL patients enrolled in ongoing clinical trials (NCT03704714 and NCT03038672). Among the five patients treated with anti-PD-1 monotherapy, three patients achieved either a partial or complete response, while two patients exhibited progression. Furthermore, Tabanelli et al. conducted an ancillary study on three patients from the Griffin et al. cohort. They investigated the immunohistochemical expression of TCF1, a transcription factor potentially associated with favorable responses to immunotherapy, in tumor biopsy specimens. Notably, the two patients who responded positively to anti-PD-1 therapy (one PR, one CR) displayed higher levels of TCF1 compared to non-responders [7]. The inflammatory microenvironment of the responders showed a greater proportion of TCF1-positive T cells (29 % in both) compared to that of the non-responder (13 %). Monitoring TCF1 positivity in THRLBCL at baseline and post-treatment with ICIs could be valuable for assessing its potential as predictive biomarker. Understanding the mechanisms of resistance and identifying biomarkers predictive of response are crucial for optimizing the use of ICIs in this lymphoma subtype.

## 3 CAR T-cell therapy in THRLBCL

CAR T-cell therapy has transformed the landscape of treatment options for R/R DLBCL [11]. Studies demonstrated high response rates and long-lasting remissions, significantly improved patient outcomes [12,13]. However, the effectiveness of CAR T-cell therapy in THRLBCL has been markedly less impressive. Nair et al.'s study shed light on the limited effectiveness of CAR T-cell therapy in patients with THRLBCL [14]. They observed a significantly lower objective response rate compared to other DLBCL subtypes, with a high proportion of patients experiencing primary resistance (not responding initially) and early relapse. In a recent study, Fein et al. conducted a retrospective analysis of data from the Center for International Blood and Marrow Transplant Research (CIBMTR) to evaluate the efficacy of CAR T-cell therapy in THRLBCL [15]. While 30 % of patients achieved progression-free survival at 2 years, the majority of patients relapsed (69 % at 2 years). Remarkably, 45 % (n = 26) of the patients had primary refractory disease, and the majority (67 %) had early disease recurrence. The largest analysis of CD19-targeted CART for R/R THRLBCL found a 2-year progression-free survival rate of approximately 30 % among patients receiving CAR T-cell. Although a significant number of patients experienced disease progression, these findings suggest that CAR T-cell therapy may provide sustained benefits for a certain group of patients with this type of lymphoma [16]. These results emphasize the need for more research into strategies for achieving durable remissions and overcoming early relapse mechanisms in patients with THRLBCL.

The upregulation of the PD-1/PD-L1 pathway constitutes pivotal mechanism underlying resistance to CAR T-cell therapy in THRLBCL patients. The high PD-L1 expression on tumor-associated macrophages and tumor cells creates an immunosuppressive environment that hinders the effectiveness of CAR T-cells [17,18]. This interaction not only inhibits the function of endogenous T-cells but also affects adoptively transferred CAR T-cells, leading to their dysfunction and diminished therapeutic response. The study by Trujillo et al. provided substantial evidence of this mechanism, showing that PD-1 was highly expressed on CAR T-cells, particularly at peak expansion phases, indicating that the immune checkpoint pathway plays a crucial role in mediating resistance [17]. Another contributing factor to the primary resistance observed in THRLBCL is the high baseline metabolic tumor volume (MTV). High MTV is associated with an increased tumor burden, which poses significant challenges for CAR T-cell therapy [17]. The extensive tumor mass not only creates a physical barrier that CAR T-cells must overcome but also contributes to an immunosuppressive microenvironment. Studies across various lymphoma subtypes, including THRLBCL, have demonstrated that a high tumor burden is correlated with reduced efficacy of CAR T-cell therapy [19].

Loss of CD19 antigen expression on tumor cells is another possible mechanism underlying resistance to CD19-directed CAR T-cell therapy in THRLBCL. Since CD19 is the target antigen for CAR T cells, its loss could make the treatment ineffective. Although the loss of CD19 antigen is relatively uncommon in DLBCL, it has been reported in some cases of early relapse following CAR T-cell therapy [19]. In the study by Trujillo et al., CD19 expression was preserved in all assessable cases of THRLBCL at progression [17]; however, the possibility of CD19 loss contributing to resistance cannot be entirely ruled out, especially given the heterogeneous nature of lymphoma biology.

## 4 Addressing resistance: future directions

**Fig. 1:**
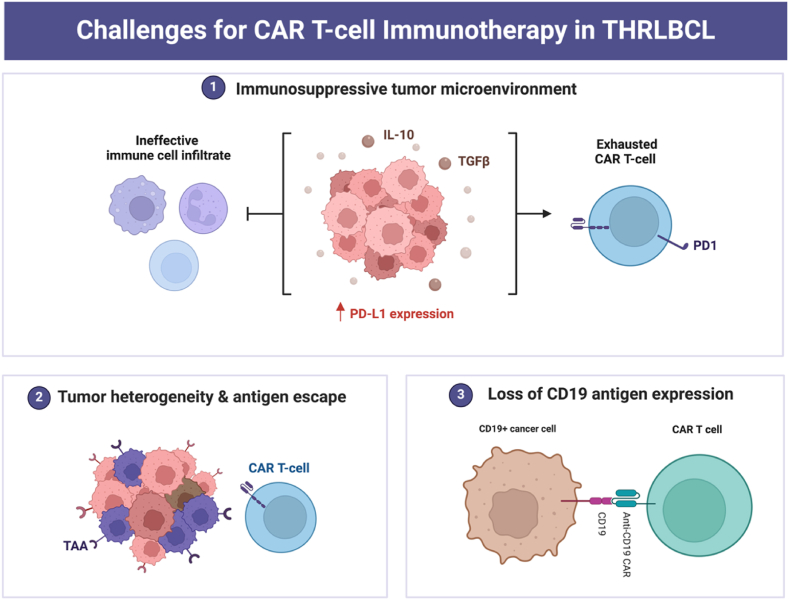
Challenges for CAR-T cell therapy in T-cell/histiocyte-rich Large B-cell Lymphoma (Created with BioRender.com.).

Overcoming resistance mechanisms remains paramount to improving the efficacy of CAR T-cell therapy in THRLBCL (Fig. 1). A promising strategy involves the combination of CAR T-cell therapy with ICIs. By targeting the PD-1/PD-L1 pathway, ICIs can potentially counteract the immunosuppressive environment and improve the function of CAR T cells. Studies have demonstrated that combining ICIs with CAR T cells or using CD19-CAR T cells engineered to express a PD-1/CD28 chimeric-switch receptor can significantly reduce tumor growth in PD-L1 positive lymphoma cells [20]. Furthermore, Chong et al. propose that PD-1 blockade with ICIs may still provide benefits for some patients with B-cell lymphomas who exhibit resistance to primary CAR T-cell therapy [21]. Interestingly, these responding patients exhibited higher expression of TCF1, potentially aligning with Tabanelli et al.'s findings and supporting the potential of TCF1 as a predictive biomarker [7]. In this context, monitoring TCF1+ exhausted T cell precursors could aid in refining patient selection for combination therapy involving ICIs and CAR T-cells.

Another potential strategy is the use of novel CAR constructs designed to target multiple antigens or incorporate signaling domains that enhance CAR T-cell persistence and resistance to exhaustion. By recognizing two different antigens, dual-targeting CAR T-cells may reduce the likelihood of antigen escape and enhance the overall efficacy of the therapy. Additionally, incorporating costimulatory domains that promote T-cell survival and proliferation can enhance the persistence of CAR T-cells in the hostile tumor microenvironment of THRLBCL.

Table 1 summarizes ongoing immunotherapy clinical trials, including patients with THRLBCL. The trials explore various combinations of CAR T-cell therapies with ICIs and other agents, aiming to improve treatment efficacy and overcome resistance. For example, one trial investigates Pembrolizumab plus CAR T-cell therapy, while additional studies investigate adding Bruton tyrosine kinase (BTK) inhibitors to CAR T-cell therapy. These trials are at various stages of development, ranging from actively recruiting participants to ongoing but not currently enrolling new patients. The primary outcomes in these trials typically focus on overall survival and complete response rates.

**Table 1:** Ongoing immmunotherapy clinical trials in THRLBCL patients.

Title	Clinical Trial No	No of patients	Location	Study Design	Status	Primary Outcome
Pembro Plus CAR T-cell Therapy in R/R in PMBCL	NCT05934448	35	USA	Interventional	Recruiting	Complete Response (CR) Rate at 6 Months
A Phase 3 Trial of Epcoritamab vs Investigator's Choice Chemotherapy in R/R DLBCL (EPCORE DLBCL-1)	NCT04628494	552	USA	Interventional	Active, not recruting	OS
Nivolumab With or Without Varlilumab in Treating Patients With Relapsed or Refractory Aggressive B-cell Lymphomas	NCT03038672	52	USA	Interventional	Active, not recruting	Overal Response Rate
Zanubrutinib and CAR T-cell Therapy for the Treatment of Recurrent or Refractory Aggressive B-cell Non-Hodgkin's Lymphoma or Transformed Indolent B-cell Lymphoma	NCT05202782	24	USA	Interventional	Recruiting	Change in 6-month complete response rates
Acalabrutinib + Liso-Cel In R/R Aggressive B-Cell Lymphomas	NCT05583149	27	USA	Interventional	Recruiting	Complete response rate (CRR)

## 5 Conclusion

Despite the success of CAR T-cell therapy in various lymphoma subtypes, THRLBCL presents a distinct challenge with a high rate of treatment resistance. The unique tumor microenvironment of THRLBCL, characterized by extensive immune infiltration and elevated PD-L1 expression, has led to the poor outcomes with CAR T-cell therapy. Combining CAR T-cell therapy with ICIs and developing dual-targeting CAR T-cells are promising avenues for further investigation. Further clinical trials are crucial to establish effective treatment strategies for this aggressive lymphoma subtype.

## Funding

The authors did not receive support from any organization for the submitted work.

## CRediT authorship contribution statement

**Taha Koray Sahin:** Writing – review & editing, Writing – original draft, Visualization, Data curation, Conceptualization. **Serkan Akin:** Writing – review & editing, Writing – original draft, Visualization, Data curation, Conceptualization.

## Declaration of competing interest

The authors declare that they have no known competing financial interests or personal relationships that could have appeared to influence the work reported in this paper.
